# Urinary tract infections in children after renal transplantation

**DOI:** 10.1007/s00467-007-0690-0

**Published:** 2009-06-01

**Authors:** Ulrike John, Markus J. Kemper

**Affiliations:** 1 University Children’s Hospitals, Kochstr. 2, 07745 Jena, Germany; 2 Klink für Kinder–und Jugendmedizin, Martinistr. 52, 20246 Hamburg, Germany

**Keywords:** Kidney transplantation, Urinary tract infection, Transplant pyelonephritis, Immunosuppression, Vesicoureteric reflux, Neurogenic bladder

## Abstract

Urinary tract infections (UTI) after pediatric kidney transplantation (KTX) are an important clinical problem and occur in 15–33% of patients. Febrile UTI, whether occurring in the transplanted kidney or the native kidney, should be differentiated from afebrile UTI. The latter may cause significant morbidity and is usually associated with acute graft dysfunction. Risk factors for (febrile) UTI include anatomical, functional, and demographic factors as well as baseline immunosuppression and foreign material, such as catheters and stents. Meticulous surveillance, diagnosis, and treatment of UTI is important to minimize acute morbidity and compromise of long-term graft function. In febrile UTI, parenteral antibiotics are usually indicated, although controlled data are not available. As most data concerning UTI have been accumulated retrospectively, future prospective studies have to be performed to clarify pathogenetic mechanisms and risk factors, improve prophylaxis and treatment, and ultimately optimize long-term renal graft survival.

## Introduction

Kidney transplantation (KTX) is now considered the treatment of choice for end-stage renal disease (ESRD) in children [1]. Patient survival is approaching 100%, and graft survival has improved significantly in recent years, mainly due to improved immunosuppressive strategies [2]. The main problem, however, chronic allograft nephropathy (CAN), remains unresolved. In CAN, specific immunological but also nonimmunological risk factors, such as hypertension and urinary tract infections (UTI), seem to play a role [3, 4], so that the ultimate solution to the problem will rely on several approaches.

Febrile UTI play a crucial role in progression of chronic kidney disease even before KTX and is often associated with anatomical urogenital abnormalities. Also, after KTX, UTI may lead to kidney damage, negatively affecting long-term graft survival by scarring and interstitial injury [5–7], although precise data for the pediatric population are scarce [8–10]. UTI management in such patients undoubtedly is more complex compared with UTI in otherwise healthy children, so that extrapolation of data is not always possible. Furthermore hardly any current diagnostic and/or treatment practices in such patients are evidence-based.

The aim of this teaching article is to summarize the clinical relevance and details of UTI after renal transplantation. Our focus is mainly on febrile UTI (fUTI), although afebrile UTI is also discussed. We discuss specific pathogenetic risk factors as well as options for treatment and prophylaxis of this frequent complication after KTX.

## Diagnosis of UTI after KTX

In general, UTI diagnosis in healthy children and those with urinary tract malformations or after KTX should follow the same principles. However, such patients after renal transplantation are more complex, as they are immunocompromised, often have anatomical urinary tract abnormalities, and maintenance of organ function is pivotal.

UTI diagnosis is confirmed with growth of 10^5^ colonies of bacteria in a collected sample. As in the case in healthy children, the method of urine collection is important in transplant recipients. Bagged specimens are unreliable due to the high degree of contamination, and midstream and catheterized urine samples are preferred in infants and older children, depending on clinical status. Suprapubic tap remains the most accurate method with the least contamination but is not used much in clinical transplantation [11, 12]. It is the only method that can exclude bacterial or fungal contamination with certainty, however. An alternative, though invasive method, of obtaining bladder urine with minimal contamination is by means of catheterization. Introduction of infection is feasible by this method, trauma of the urethra may occur, and psychological stress for patient (and doctor) may be high. The method may be superior in the ill child, however, when urinalysis is required quickly, especially when bladder volume is low [11].

Urinary abnormalities in UTI include leukocyturia, hematuria, and positive leukocyte esterase, often identified by dipstick testing. The presence of white blood cells (WBC) in urine is not limited to UTI but may occur in other conditions, such as acute rejection, BK nephropathy, and presence of foreign material, such as a stent, in the urinary tract. Data on the diagnostic impact of these tests in KTX are not available.

In fUTI after KTX (transplant pyelonephritis), elevation of inflammatory markers are typical, such as leucocytosis, erythrocyte sedimentation rate, and C-reactive protein (CRP). These parameters are not specific for UTI, but they may be important to distinguish between transplant pyelonephritis and rejection episodes [13], as renal dysfunction is a typical feature for both. They are also important in follow-up, as they are tools for monitoring response to antibiotic treatment [14].

**Fig. 1:**
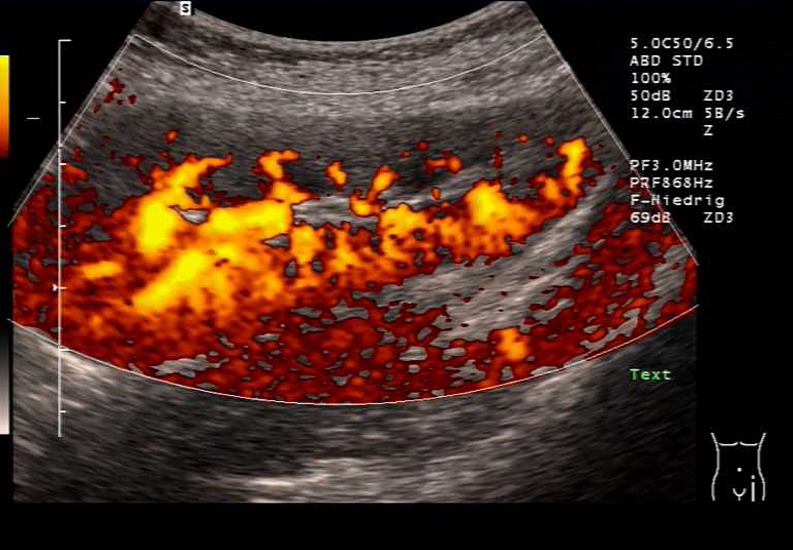
Longitudinal power Doppler ultrasonogram of a patient with transplant pyelonephritis showing vascular hypoperfusion in the cortex

**Fig. 2:**
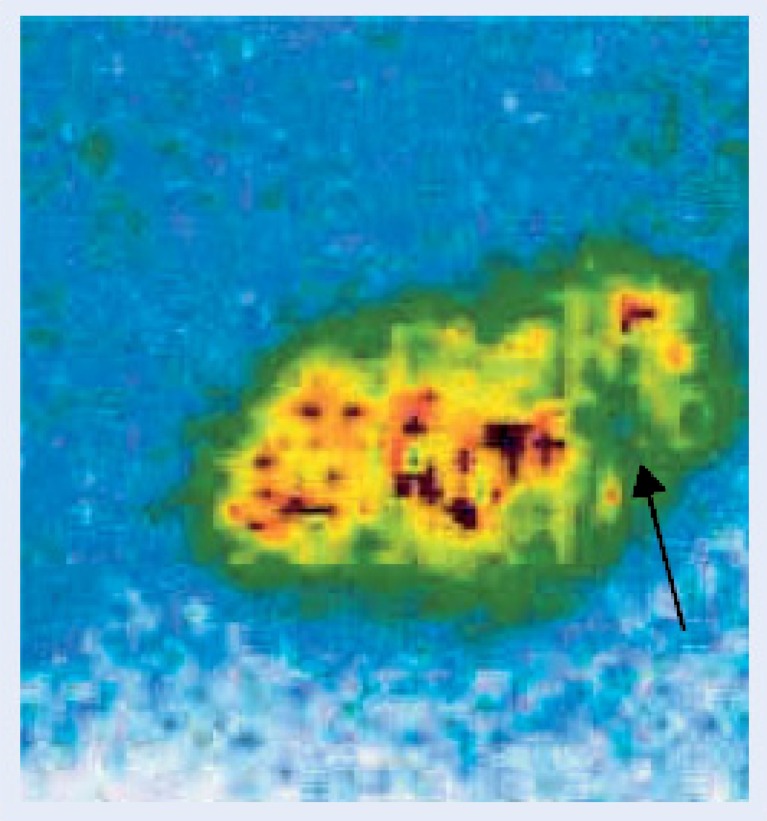
99mTc DMSA scan of a patient with acute transplant pyelonephritis showing an uptake defect in the lower pole (arrow)

There are few data concerning the optimal diagnostic imaging during fUTI after KTX. Ultrasound is a noninvasive tool and may demonstrate hydronephrosis or swelling of the transplanted kidney. Perfusion deficits may be visualized by color-Doppler ultrasound, especially in the power mode (Fig. 1). Dimercaptosuccinate acid (DMSA) scintigraphy is regarded as the gold standard in the diagnosis of acute pyelonephritis as well as in the documentation of residual scarring. This tool has been employed only occasionally in UTI after renal transplantation (Fig. 2). Long-term data suggest that fUTI may lead to focal defects on DMSA scanning [5, 15], but also other events, such as vascular complications and renal biopsies, may do so, which has to be considered in the interpretation of this diagnostic tool [16]. However, at present, no clear recommendations can be made as to the use of DMSA scanning as a diagnostic tool in transplant pyelonephritis, neither in the acute phase, nor in follow-up.

## Classification of UTI after KTX

Most importantly, afebrile UTI must be distinguished from fUTI. Afebrile UTI may also affect the native kidneys, which is a diagnostic dilemma. Patients with afebrile UTI after KTX may be symptomatic (dysuria, frequency, etc.) or may be completely asymptomatic, raising the question of whether treatment is indicated. Furthermore, UTI, both febrile and afebrile, may show recurrences. It should be noted that immunosuppression, especially with steroids, might alter the temperature response to infections by suppressing cytokine production.

## Clinical features

Fever and renal dysfunction are the typical features of fUTI after KTX. In some patients, symptoms and signs of urosepsis may develop. The acute deterioration of renal function during UTI is a well-known feature [6, 17] which, together with the significant inflammatory parenchymal response, emphasizes the potential risk of tissue damage in the transplanted kidney in acute and long-term follow-up, despite normalization of serum creatinine values after UTI. Acute rejection episodes may be triggered by fUTI [18], and development of an intrarenal abscess in the graft following UTI has also been described [19].

## Prevalence

A high prevalence of fUTI in children after renal transplantation has been demonstrated by several retrospective studies. They are not limited to the immediate posttransplant period but also occur later, especially in girls [14]. In pediatric studies, the prevalence ranged between 15% and 33% [6, 9, 10, 17]. Higher prevalence of up to 61% has been demonstrated in adults; however, in some studies, entry criteria were less strict, such as inclusion of patients with (asymptomatic) afebrile bacteriuria [20, 21]. Recently, Pelle et al. demonstrated a prevalence of UTI of 75.1%; 18.7% of these patients developed transplant pyelonephritis [22]. Other studies have found a higher frequency in children; for instance, admissions for UTIs in pediatric patients after renal transplants in the United States averaged nearly 150 per 1,000 patient years at risk, compared with 67 among adults (editorial comment following [23]).

## Epidemiology

Although *Escherichia coli* remained the most frequently isolated microorganism, as in other studies [8, 24, 25], it was isolated less frequently than in the general pediatric population, where it is found in up to 80% of UTIs [26]. This may be due to the underlying immunosuppression and colonization. Also, some patients receive antibiotic prophylaxis or treatment, which may have an impact on colonization. Surveillance urinary cultures may help, especially for patients with recurrent UTI and those with abnormal lower urinary tract and neurogenic bladder; not infrequently, bacteria with multiple antibiotic resistances, such as *Pseudomonas* sp., can be detected. Table 1 demonstrates epidemiology of fUTI in published series as well as in control populations.

**Table 1:** Epidemiology of febrile urinary tract infections (fUTI) after kidney transplantation (KTX). Due to rounding, numbers may not add up to exactly 100%

Epidemiology of fUTI following renal transplantation in selected studies									
Microorganism	John [14]	Mueller [6]	Valera [47]	Alangaden [48]	Pellè [22]	Chuang [8]	Mahara [20]	Pape [49]	Haller [46]
Patients with UTI	40/110	15/47	41/161	60/127	133/177	213/500	103/192	100	684
Age group	Children	Children	Mostly adults (84%)	Adults	Adults	Adults	Adults	Nontransplant^a^	Nontransplant^b^
Episodes of UTI (*n*)	*56*	*35*	*58*	*60*	*595*	*213*	*173*	*100*	*684*
Gram-negative bacteria (%)									
*Escherichia coli*	37	46	71	21	28	29	38	47	57
*Klebsiella*	4	3	5	15	3	10	8	4	5
*Proteus*	4				0			8	6
Other Enterobactericae	6	9			6		13		4
*Pseudomonas aeruginosa*	13	6	10	10	15	4	7	5	7
Gram-positive bacteria (%)									
*Staphylococcus aureus*		3	3			12	1		
*Enterococcus* spp.	15		3	33	24	24	26	23	14
*S. epidermidis*	4				12		4		
*Streptococci*	2		3		3	4			
Fungal (%)						5			
*Candida*			1				2		
Other (%)	15	34	4	21	9	12		13	6
Total	100	101	100	100	100	100	99	100	99

^a^Community-acquired UTI, excluded: renal diseases, anatomic abnormalities of the urinary tract, and recurrence

^b^Evaluation did not consider whether UTI was complicated or uncomplicated, the first or recurrent infection, or nosocomial or community-acquired

## Risk factors

Several risk factors for UTI have been elucidated by studies in adults and children. Knowledge of these risk factors is one key for prevention.

1. Anatomical factors
  Underlying urological abnormalities have to be regarded as risk factors for UTI even before transplantation, such as hydronephrosis, vesicoureteric reflux (VUR), and/or neurogenic bladder, often in combination. Surgery of the urinary tract before listing for transplantation is often necessary and should decrease the risk of infections, although in our retrospective study, operated children per se had more UTIs, possibly because patients represent a specific population at risk, even after surgery [14]. Patients with urinary tract malformations compromise about a third of children with end-stage renal failure and include heterogeneous disorders such as posterior urethral valves, prune-belly syndrome, spina bifida, or urogenital sinus abnormalities, which may lead to lower urinary tract dysfunction [27–30]. In our study, structural disorders and neurogenic bladder were present in 33% of all patients with fUTI.
  Especially structural abnormalities of the bladder anatomy and neurogenic bladder have been regarded as specific risk factor that needs to be distinguished from behavioral voiding problems (voiding dysfunction). Neurogenic bladder leads to a higher incidence of UTI, with associated morbidity and poorer graft function [31]. The presence of focal scars was associated with either raised intravesical pressures or recurrent UTIs [5]. Although no controlled data are available that bladder augmentation procedures prevent or decrease the rate of UTI after transplantation, it seems mandatory that evaluation and treatment of neurogenic bladder is very important and should be performed before renal transplantation, requiring specific follow-up after KTX.
2. Functional factors
  In addition to abnormal bladder anatomy and neurogenic bladder, voiding dysfunction is present in many patients and may also increase the risk of UTI in analogy to the nontransplant population [32]. On the other hand, a recent retrospective study from Herthelius et al. [33, 34] suggested no increased frequency of UTI after KTX in children with bladder dysfunction. However, the study did not analyze the effect of interventions in the patients with voiding dysfunction.
3. Vesicoureteric reflux (VUR)
  Among the anatomical factors, the pathogenetic role of VUR has been discussed as an especially important risk factor for UTI after renal transplantation. A clear distinction has to be made, however. First, VUR is present in many patients as an underlying diagnosis, and UTI could affect the native and the transplanted kidney (or both). The differential diagnosis between these entities is almost impossible without sophisticated imaging studies. Some authors recommend native nephrectomy of kidneys with VUR to decrease the risk of native-kidney UTI after KTX [35]. However, no controlled studies are available. Also, pretransplant antireflux surgery did not reduce the risk for fUTI after KTX in a recent study of Basiri et al., who compared 12 children with VUR who underwent reimplantation to 17 children with VUR who did not [36]. The lack of difference may be due to dysfunctional voiding that is also often present in children with VUR and that may be an independent risk factor for UTI, of both native and transplanted kidney.
  Second, VUR into the transplanted kidney in a previously normal urinary tract may develop secondary to transplant surgery, thereby increasing the risk for transplant pyelonephritis. Dunn et al. [10] found an increased incidence of pyelonephritis in pediatric renal transplant patients who had VUR. Ranchin et al. tested 55 of 84 transplanted children during a period of 5 years [9] and demonstrated a 58% prevalence of VUR. The rate of UTI was 60% and was similar in patients with or without VUR in the overall analysis. However, after excluding UTI related to catheterization, the rate of transplant pyelonephritis was higher in patients with VUR (*p* < 0.02). Whether strict antireflux surgery can reduce the risk of fUTI after renal transplantation has not been studied so far. Correction of vesicoureteric reflux into the kidney graft has been shown to reduce the incidence of UTI in a small series but was associated with obstructive complications [17], which may be high, especially in the cohort with associated abnormal bladder anatomy.
4. Stents and other manipulations
  Foreign material, such as stents, urinary catheters, and suture material, can cause UTI, as it is frequently colonized by bacteria (and fungi). In the study by Kamath et al., the odds ratio for UTI for ureteric stents was 4.6 [37]. Therefore, these devices should only be used for a short time [20] or avoided if possible. Manipulation with or insertion of such foreign material should therefore be performed with caution and possibly with antibiotic prophylaxis, especially in the initial posttransplant period, when high-dose immunosuppression increases the risk even further.
5. Gender
  The rate of UTI in adult females has been reported to be twice as high as in male renal transplant recipients [8, 20, 38], and we were able to confirm this finding for children in our retrospective multicenter analysis [14]. Anatomical reasons (e.g. shortness of urethra) may be relevant in children and adults, and in female adolescents, sexual activities have to be taken into account (and asked about), as well as predisposing immunological factors in females [39].
6. Immunosuppression
  Immunosuppression has an impact on defense mechanisms so that a theoretical impact on the incidence of UTI should be present. A recent study indicated an increased risk for patients on a mycophenolic-based regimen, with an odds ratio of 1.9 [37], but not for other immunosuppressants, especially induction treatment with anti-CD3. Therefore, patients on high-dose immunosuppression should be regarded as at risk for bacterial and viral infections, including UTI. An accumulation of risk factors (e.g. unnecessary catheterization or manipulation at the urinary tract, should be avoided or at least limited. Future treatment studies should, in detail, also look at the impact of specific immunosuppressants on infection, including UTI.
  In our opinion, it should be emphasized that a combination of risk factors occurs in many patients and that it is unlikely that a single factor is responsible for the development of febrile or afebrile UTI.

### Do urinary tract infections affect long-term outcome?

Several reports indicate that despite acute graft dysfunction during fUTI, long-term renal function was not different between patients with and without infection [14, 40, 41]. Other reports show a less optimistic picture in patients with UTI, not only in adults [22], but especially in children [9, 34], and even more for those with recurrent UTIs [34]. In this respect, especially the retrospective cohort study of Abbott et al. in nearly 29,000 adult renal transplant patients of the United States Renal Data System (USRDS) database is of interest, because late UTIs were independently associated with the risk of subsequent graft loss and even death [38]. It remains difficult, however, to properly assess the risk in these uncontrolled and partially selective series, especially in view of an imperfect endpoint (i.e. serum creatinine). It is also of special note that also in asymptomatic (afebrile) UTI, serum creatinine increases have been reported [24].

More studies into the pathophysiology of UTI after KTX are necessary in this respect. Serotypes and adherence factors of bacteria such as *Escherichia coli* may have a strong impact on acute and long-term graft dysfunction, as suggested by a recent study from Rice et al. [42]. The intensive inflammatory response indicated by CRP or other markers, such as procalcitonin response, may be related to the degree of tissue destruction leading to fibrosis [43]. The concurrent treatment with steroids with their anti-inflammatory properties may possibly even be a protective factor [44]. All these issues need to be addressed to clarify the issue of long-term endangerment. Last but not least, long-term clinical variables need to be defined and collected.

### Treatment of UTI after renal transplantation

Aggressive and specific treatment of fUTI is mandatory. In our opinion, in fUTI, parenteral antibiotics should be preferred, at least initially, to achieve fast tissue saturation. The optimal treatment duration has not been studied, but we favor 14 days in transplant pyelonephritis. In accordance with Benador et al., we sometimes continue with oral treatment when the clinical situation has improved [45]. As *Enterococcus* and *Pseudomonas* spp. are more frequent, we currently use a combination of ceftazidime and ampicillin to cover for *E. coli, Pseudomonas*, and *Enterococcus*. Others have recommended ampicillin and gentamicin for the same reason [46]. However, nephrotoxicity of the latter is a concern. Oral fluoroquinolone medications such as ciprofloxacin is another alternative in this situation. Fungal UTIs may occur and require specific treatment; in our center, antifungal prophylaxis is given during high-dose antibiotic treatment. Treatment recommendation for other centers may be different, however. Monitoring of blood levels is important during acute UTI, as antibiotic treatment may interfere with resorption. Steroid dose must be increased sometimes during fUTI, especially early after renal transplantation, to avoid symptoms of adrenal insufficiency.

Symptomatic afebrile UTI may be treated with oral antibiotics unless specific risk factors are present (renal dysfunction etc.) [11]. Again, treatment should be specific and oral cephalosporin may be the first choice, especially if the patient was on antibiotic prophylaxis. Whether asymptomatic UTIs have to be treated remains controversial and is often an individual decision. In patients with abnormal bladder anatomy and catheterization, such as in spina bifida, colonization is frequent, and symptoms such as dysuria may be absent. There is no evidence and no consensus as to whether in these patients bacterial colonization needs treatment, including bladder washing with antibiotics. In our centers, we currently only treat symptomatic patients with abnormal bladder anatomy and bacterial colonization and do not use antibiotic bladder irrigation.

### Practical consequences for the clinician

Although UTIs cannot be prevented completely, preventative measures may be able to reduce the incidence and lead to earlier and more effective treatment. Recurrences of fUTI should be prevented, if at all possible.

1. Antibiotic prophylaxis
  There are no systematic studies relating to use of prophylactic antibiotics after renal transplantation. They may be useful in the view of the high prevalence of UTI, however, and most pediatric transplant centers prescribe prophylactic antibiotics for the first 3–6 months posttransplantation, or longer in refluxive patients [31, 41], but sometimes also for *Pneumocystis carinii* prophylaxis. However, not all studies confirmed a beneficial effect and even demonstrated a high bacterial resistance rate [24]. If prophylactic antibiotics are used, it seems that they should be administered for prolonged periods, as fUTI may occur late, especially in girls.
2. Bladder dysfunction
  As bladder dysfunction contributes to the risk of UTI, exact diagnosis (and treatment) before transplantation and surveillance is important. Simple methodology (micturition protocols, uroflowmetry, and bladder ultrasound) for this evaluation exists and needs to be used. Sometime, however, it is not possible to evaluate bladder function before transplantation, e.g. in the anuric patient who never had a voiding history. Patients with structural and functional bladder abnormalities require regular urodynamic monitoring and treatment: pharmacologic, behavioral, or surgical. This includes frequent voiding, treatment of constipation, and sometimes intermittent catheterization. Correction of structural anomalies and optimization of storage and emptying functions of the bladder is recommended before transplantation. However, an abnormal urinary bladder is no longer a contraindication to renal transplantation [21, 28, 31].
3. Miscellaneous
  Other measures include high oral fluid intake and hygienic measures, especially in the adolescent, sexually active patients. Sometimes, home testing of urine by the parent or patient with dipsticks is helpful to detect UTI early. No data are available for alternative methods, such as cranberry juice, urine acidification, etc.

## Summary and conclusion

Children after renal transplantation are at high risk for UTIs. Although anatomical factors including neurogenic bladder increase the risk for UTI, the high prevalence in girls and in patients with nonanatomical underlying disorders indicate that further risk factors are present. The severe renal dysfunction during fUTI and inflammatory response indicates that fUTI has to be regarded as a serious complication, endangering long-term graft survival. Therefore, prophylactic measures including antibiotic prophylaxis and bladder training should be considered. Prospective studies are urgently needed to evaluate precise mechanisms and the value of preventive measures of this important complication.

## Questions

(Answers appear following the reference list)

Which answer is true: UTIs after KTX

1. *Pseudomonas* and *Enterococcus* spp. cause UTIs after renal transplantation:
  - In the same frequency as in unimmunocompromised patients
  - More frequently than in immunologically healthy children
  - Less frequently than in immunologically healthy children
  - Are specifically related to graft rejection episodes
  - None of the above is true
2. Which answer is **not** true: diagnostic workup of a patient after renal transplantation with fever typically includes:
  - Renal biopsy
  - Renal ultrasound, including Doppler ultrasonography
  - Urine culture
  - Laboratory testing, including full blood count, serum creatinine and CRP
  - Clinical examination
3. Long-term complications after fUTI in patients after KTX:
  - Are irrelevant
  - Develop only in patients with acquired diseases
  - Have been documented and include accelerated graft loss and chronic allograft nephropathy
  - Are only present in females
  - Can be prevented by specific immunosuppression
4. Which answer is **not** true: the main differential diagnosis of a patient with kidney graft dysfunction and fever includes:
  - Cytomegalovirus infection
  - Acute rejection
  - Transplant pyelonephritis
  - Dehydration during viral illness
  - Malnutrition
5. Risk factors for UTI after KTX include:
  - Underlying urinary tract pathology
  - Female gender
  - VUR into kidney transplant
  - Voiding dysfunction and constipation
  - All of the above
